# Consultation-Liaison Psychiatry During COVID-19 Lockdown: A Retrospective Chart Review

**DOI:** 10.7759/cureus.11048

**Published:** 2020-10-19

**Authors:** Shubham Jhanwar, Vijay Krishnan, Jitendra Rohilla

**Affiliations:** 1 Psychiatry, All India Institute of Medical Sciences, Rishikesh, IND

**Keywords:** covid-19, suicide, consultation-liaison psychiatry, lockdown

## Abstract

The coronavirus disease 2019 (COVID-19) pandemic has caused a major psychosocial impact in the community due to its direct effects and restrictive control strategies, e.g. lockdown. The current pandemic, a highly stressful situation, can predispose not only vulnerable but previously well-adjusted individuals for psychological disorders. A retrospective chart review of consultation-liaison psychiatry (CLP) case records was conducted for one month before and after the start of lockdown. Patients seen during lockdown were relatively younger; t = 1.8, p = 0.074. The most common psychiatric emergency was a suicidal attempt (34.3%) and delirium (35.4%) during and before lockdown, respectively. The probability of the emergency psychiatry presentation for attempted suicide increased significantly during lockdown (odds ratio (OR) 8.0, 95% CI 2.03 to 31.57, p = 0.003). The most common stressors for CLP patients with suicide attempts during lockdown were relationship issues and loss of privacy. It seems that stressors arising due to the current crisis are not only highly severe and multiple but qualitatively different. Further studies with larger sample sizes and from other parts of the country can further improve our understating of the psychological impact of the COVID-19 pandemic in the affected community. Needless to say, higher vigilance in the community for at-risk individuals, availability, and awareness about telemedicine services can play an important role to combat the risk of suicide during the lockdown.

## Introduction

The coronavirus disease 2019 (COVID-19) pandemic has caused multiple psychosocial changes in the community. A recent online survey in the Indian community through the snowball sampling technique has revealed that three-fourths of the participants felt the need for mental health care to deal with the panic of the COVID-19 pandemic [1]. A study conducted in China during the initial stage of COVID-19 found the increased prevalence of anxiety and depression among individuals presenting with physical symptoms like cough, myalgia, and sore throat [2]. More than half (53.8%) of participants reported moderate or severe degree of the psychological impact of the outbreak; 16.5% had moderate to severe depressive symptoms and 28.8% had moderate to severe anxiety symptoms. Since the first case reported in February 2020, suicide-related news is common in Indian media [3]. Suicide is a leading cause of death in the Indian population, particularly among the younger age groups [4]. Severe stress is a known precipitating factor for self-harm and suicide. However, definitive data are yet to come about the number and risk of suicide due to stress of the COVID-19 pandemic and nationwide lockdown. Ministry of Home Affairs, Government of India issued an order on March 24, 2020, to implement effective measures to prevent the spread of COVID-19 in the country. These measures were called Lockdown, during which all government offices, public corporations, autonomous bodies, commercial and private establishments, industrial establishments, transport services, educational, training and coaching institutions, and places of worship were closed except for essential services like grocery shops and medical services [5]. Any kind of gathering irrespective of purpose (social, political, sports, entertainment, cultural, religious) was prohibited during this period. This study aimed to evaluate the clinical profile of patients admitted to the emergency department with psychological problems during the lockdown and compared this data with a similar duration in the pre-lockdown period.

## Materials and methods

Samples

Chart review was conducted for Consultation-Liaison Psychiatry (CLP) case records of all patients admitted to the emergency department of a government tertiary care teaching hospital for two months from February 24, 2020, to April 23, 2020. The following information was obtained: demographic and clinical information, e.g. reason for the emergency consultation, past psychiatric history, diagnosis, history, and details of self-harm/suicide. Additional information regarding stressors leading up to the suicide attempt was collected from an on-call member of the CLP team who has personally seen patients visited the emergency department.

Statistical analysis

The data were divided into two groups, based on the date of the announcement of the lockdown in the regions (March 24, 2020). Group 1 included the patients seen by the CLP team before lockdown from February 24, 2020 to March 23, 2020. Group 2 included the patients seen by the CLP team after lockdown from March 24, 2020, to April 23, 2020. The mean age of both groups were compared with an independent sample t-test. Categorical variables were compared using the chi-square test.

## Results

Case records of 83 patients seen by the CLP team were retrieved, 51 before lockdown (Group 1) and 32 during lockdown (Group 2). Table 1 shows sociodemographic and clinical characteristics.

**Table 1:** Socio-demographic and clinical characteristics SD- Standard deviation t = Independent sample Test X^2^ = Chi-square test OR = Odds Ratio

Total patients(N=83)	Before Lockdown 51(61.44%)	During Lockdown 32(38.55%)	Test of significance	p value
All Psychiatric emergency				
Age				
Mean (SD)	36.47 (16.14)	29.90 (16.09)	t =1.807	0.074
Range	10 – 82	7- 80		
Gender				
Male	37 (72.5%)	11 (34.3%)	X^2^ = 11.75	< 0.01
Female	14 (27.5%)	21 (65.6%)		
Psychiatric emergency without suicide attempt	48 (94.11%)	21 (65.6%)	OR = 8.38 (2.12 – 33.17)	<0.01
Psychiatric emergency with suicide attempt	3 (5.88%)	11 (34.3%)		
Method of Suicide Attempt				
Hanging	0%	54.5%		
Poisoning	66.6%	27.3%		
Drug Overdose	33.3%	18.2%		
Stressor for Suicide attempt				
1. Economic lose	50%	9%		
2. Job Lose	33%	9%		
3. Relationship Issue	33%	81.8%		
3A. Family member	0	33.3%		
3B. Boyfriend/Girlfriend	100%	66.6%		
4. Loss of Privacy	0%	54.5%		

The mean age of Group 2 patients (29.9 ± 16.9) was less compared to Group 1 (36.47 ± 16.1) though the difference was not found to be statistically significant. There were more female patients in Group 2 (65.6%) compared to Group 1 (27.5%); χ2(1) = 11.75, p < 0.01.

The most common psychiatric emergency during lockdown was suicide attempt (11, 34.3%) while it was delirium (17, 35.4%) before lockdown. The proportion of suicide cases increased from three (5.88%) to 11 (34.3%). Odds of emergency psychiatric patients presenting with suicide attempt increased during lockdown; odds ratio (OR) = 8.38, CI = 2.12 - 33.17, p < 0.01. A similar increase was not seen for any other psychiatric emergency. The mean age of patients presenting with suicide attempt did not differ between the two groups; t = 0.52, p = 0.61. Similarly, there was no difference in the mean age of patients who presented with other types of psychiatric emergency; t = 0.74, p = 0.46.

Patients who presented with suicide attempt in Group 1 used the method of either a drug overdose or pesticide poisoning. In Group 2, hanging (54.5%) was the predominant method for suicide attempt during the lockdown. Two patients in Group 2 attempted suicide by consuming a large amount of sanitizer. While either economic or job loss was the major stressor in Group 1, relationship issues either with a family member or partner and loss of privacy was the predominant problem faced by patients in Group 2. The aspects of life affected by loss of privacy were personal relationship and communication with friends and partners, outside family. A majority of the emergency patients presented with suicide attempt in Group 2 reported stressors in the form of problems in a love affair, difficulty in meeting a partner, and feeling restrictions in their freedom due to being too close to family members during the lockdown.

## Discussion

The total number of emergency psychiatric cases reduced by around one-third during the lockdown compared to similar duration before lockdown. This is counter-intuitive given the relative lack of other psychiatric services (in-patient and out-patient) in both public and private health systems during the COVID-19 lockdown in India [6]. Several reasons may be considered for this. One, patients may have difficulties accessing emergency services due to travel restrictions imposed by government authorities and ban on both public and private transport medium during the lockdown in India. Patients and attendants may choose not to attend emergency services for fears of contracting the infection [7].A reduction in the rate of a referral might have occurred, as emergency physicians prioritized quicker discharges to prevent crowding and maintain a lower bed occupancy rate in the emergency department.

There was a steep increase in the number of patients presenting with suicide attempts, from three before the lockdown to 11 in the month during the lockdown, despite the reduction in overall emergency psychiatric referral. While a relative increase could be accounted for by changes in patients' and attendants' willingness to attend emergency or referral rates to psychiatry, the increase in absolute numbers is harder to explain. There is a possibility that due to the unavailability of mental health services in private hospitals or smaller government facilities, patients are being diverted to our emergency department in larger numbers. It is uncommon to see private hospitals usually unwilling to accept patients presenting acutely after a suicide attempt because of anticipated medico-legal concerns, and suicide attempts are primarily managed by medical facilities closest to the incident. State governments in India have started taking actions in this regard to reduce the load on the government health system [8].

There is some support for the consideration that the incidence of suicide attempts has increased in the lockdown period. These include studies conducted during the COVID-19 period as well as in previous crises [9-10]. There are also findings from other studies that show that the proportion of relatively severe psychiatric presentations may have increased as mental illnesses remain untreated in the community for longer periods, leading to presentations with greater morbidity. Thus, a proportion of these presentations might represent the widening mental health treatment gap as a consequence of the lockdown.

Due to the low number of suicide attempts included for the pre-lockdown period, the presentations in the post-lockdown period are best compared to nationally representative data for characteristics of suicide. Here we see a relative preponderance of female suicide attempts, which is in line with national data, and hanging as the predominant method, even amongst female subjects [11]. This is an important finding for two reasons. In the first place, most Indian data consistently shows chemical overdoses (medications, pesticides) as a more commonly used mode of suicide by women than men [11]. Secondly, hanging is a method that may have higher lethality as compared to overdoses [12].The increased number of suicide attempts by hanging during the lockdown period could be due to limited access to other methods and means of self-harm in view of various restrictions imposed. For example, patients could not obtain pesticides or other chemicals because of closure of shops and transport services. 

The study also found that economic loss and job loss were predominant stressors in suicide cases before lockdown, but during the lockdown, love affairs and loss of privacy were the most common stressors for a suicide attempt. During COVID-19, both government and private employers allowed their workers to work from home as well as the financial support provided from the state during the lockdown. In the above scenario, all members of the family were spending most of the time together with more face-to-face time. This supports a higher proportion of relationship issues and loss of privacy as the predominant stressors during the lockdown period seen among emergency psychiatry patients in this study.

Limitations

In view of the short duration of the study, there is a possibility of not including the psychiatry disorders with delayed/insidious onset. Some cases of mild severity particularly anxiety disorder may not be seen by CLP team because of various reasons like a primary team not referring and handling the case itself and early discharge of non-COVID patients from an emergency to minimise the spread of infection of coronavirus.

## Conclusions

Large-scale community-based data regarding psychosocial impact due to COVID-19 is still awaited in India. The findings of our study point to the possibility of both quantitative and qualitative changes in psychiatric conditions requiring a visit to the emergency department for a suicide attempt during the lockdown. As the COVID-19 pandemic continues to affect the Indian population, mental health services may be extended to help mitigate its psychological impact on the community. A large proportion of subjects attempting suicide feel helpless and hopeless before the attempt. Though not face-to-face, telepsychiatry can make the vulnerable individual feel that they have been 'listened', 'understood', and 'their distress acknowledged'. A short-term training program for community health workers in the early identification of vulnerable individuals will be useful in this crisis.

## References

[1] Roy D, Tripathy S, Kar SK, Sharma N, Verma SK, Kaushal V (2020). Study of knowledge, attitude, anxiety & perceived mental healthcare need in Indian population during COVID-19 pandemic. Asian J Psychiatr.

[2] Wang C, Pan R, Wan X (2020). Immediate psychological responses and associated factors during the initial stage of the 2019 coronavirus disease (COVID-19) epidemic among the general population in China. Int J Environ Res Public Health.

[3] Goyal K, Chauhan P, Chhikara K, Gupta P, Singh MP (2020). Fear of COVID 2019: first suicidal case in India !. Asian J Psychiatr.

[4] Patel V, Ramasundarahettige C, Vijayakumar L (2012). Suicide mortality in India: a nationally representative survey. Lancet.

[5] (2020). Circulars For Covid-19. https://www.mha.gov.in/notifications/circulars-covid-19.

[6] Hiremath P, Suhas Kowshik CS, Manjunath M, Shettar M (2020). COVID 19: Impact of lock-down on mental health and tips to overcome. Asian J Psychiatr.

[7] Mauro V, Lorenzo M, Paolo C, Sergio H (2020). Treat all COVID 19-positive patients but do not forget those negative with chronic diseases. Intern Emerg Med.

[8] Banerjee S (2020). Private hospitals refusing to treat COVID-19 patients to face action. https://www.thehindu.com/news/cities/mumbai/private-hospitals-refusing-to-treat-covid-19-patients-to-face-action/article31727178.ece.

[9] Hooper R (2020). How the coronavirus crisis is affecting your dreams. New Sci.

[10] Cheung YT, Chau PH, Yip PS (2008). A revisit on older adults suicides and Severe Acute Respiratory Syndrome (SARS) epidemic in Hong Kong. Int J Geriatr Psychiatry.

[11] (2020). Accidental deaths and suicides in India. New Delhi: Government of India. http://ncrb.gov.in/StatPublications/ADSI/ADSI2015/chapter/2%20suicides-v1.pdf.

[12] Moreno C, Wykes T, Galderisi S, Nordentoft M, Crossley N, Jones N (2020). How mental health care should change as a consequence of the COVID-19 pandemic. Lancet Psychiatry.

